# Forced Desynchrony Reveals Independent Contributions of Suprachiasmatic Oscillators to the Daily Plasma Corticosterone Rhythm in Male Rats

**DOI:** 10.1371/journal.pone.0068793

**Published:** 2013-07-22

**Authors:** Cheryl Wotus, Travis R. Lilley, Adam S. Neal, Nicole L. Suleiman, Stefanie C. Schmuck, Benjamin L. Smarr, Brian J. Fischer, Horacio O. de la Iglesia

**Affiliations:** 1 Department of Biology, Seattle University, Seattle, Washington, United States of America; 2 Department of Biology, University of Washington, Seattle, Washington, United States of America; 3 Program of Neurobiology and Behavior, University of Washington, Seattle, Washington, United States of America; 4 Department of Mathematics, Seattle University, Seattle, Washington, United States of America; Nagoya University, Japan

## Abstract

The suprachiasmatic nucleus (SCN) is required for the daily rhythm of plasma glucocorticoids; however, the independent contributions from oscillators within the different subregions of the SCN to the glucocorticoid rhythm remain unclear. Here, we use genetically and neurologically intact, forced desynchronized rats to test the hypothesis that the daily rhythm of the glucocorticoid, corticosterone, is regulated by both light responsive and light-dissociated circadian oscillators in the ventrolateral (vl-) and dorsomedial (dm-) SCN, respectively. We show that when the vlSCN and dmSCN are in maximum phase misalignment, the peak of the plasma corticosterone rhythm is shifted and the amplitude reduced; whereas, the peak of the plasma adrenocorticotropic hormone (ACTH) rhythm is also reduced, the phase is dissociated from that of the corticosterone rhythm. These data support previous studies suggesting an ACTH-independent pathway contributes to the corticosterone rhythm. To determine if either SCN subregion independently regulates corticosterone through the sympathetic nervous system, we compared unilateral adrenalectomized, desynchronized rats that had undergone either transection of the thoracic splanchnic nerve or sham transection to the remaining adrenal. Splanchnicectomy reduced and phase advanced the peak of both the corticosterone and ACTH rhythms. These data suggest that both the vlSCN and dmSCN contribute to the corticosterone rhythm by both reducing plasma ACTH and differentially regulating plasma corticosterone through an ACTH- and sympathetic nervous system-independent pathway.

## Introduction

Activation of the hypothalamic-pituitary-adrenal (HPA) axis and the ultimate release of glucocorticoids (GCs) under conditions of stress is an adaptive response that is critical for the survival of all vertebrates. In addition to its homeostatic response to stress, the HPA axis exhibits a characteristic daily rhythm, with peak GC secretion occurring prior to the onset of activity (i.e. morning for humans, and evening for nocturnal animals, like rats). This coinciding GC rise with the onset of activity aids in preparing the organism for the increased energetic demands of wake relative to sleep.

The daily rhythm in HPA activity is primarily driven by the brain’s endogenous pacemaker, the suprachiasmatic nucleus (SCN) of the hypothalamus [1,2]. The SCN has been shown to modulate GC release, in part, by controlling the release of corticotropin-releasing hormone (CRH) from neurons in the paraventricular nucleus (PVN) of the hypothalamus, which in turn induces the release of the GC primary secretagogue, adrenocorticotropic hormone (ACTH), from the anterior pituitary gland. Initial studies in rats showed that the SCN was responsible for inhibiting HPA activity at the nadir of the rhythm through vasopressinergic input to neurons in the dorsal medial nucleus of the hypothalamus (DMH), which in turn project to parvocellular regions of the PVN [3–5]. Further studies have also suggested that the evening rise in HPA activity may be the result of stimulatory influences from the SCN via the release of vasoactive intestinal peptide (VIP) and possibly other transmitters [6].

The evening rise in GCs tends to be higher in amplitude than that of ACTH; in fact, rhythms in plasma ACTH are sometimes undetectable [6–10]. This apparent dissociation between changes in ACTH and corticosterone has led to the hypothesis that the diurnal variation in plasma corticosterone is also due, in part, to changes in adrenal sensitivity to ACTH and/or ACTH-independent modulation of the adrenal. There is evidence that sympathetic innervation of the adrenal gland through the thoracic splanchnic nerve contributes to the regulation of circadian changes in corticosterone secretion by affecting adrenal sensitivity [11–13]. It has been speculated that such neural regulation could be directly controlled by the SCN, through input to neurons of the autonomic nervous system in the PVN that send descending projections to the spinal cord [14]. This multisynaptic neural pathway from the SCN to the adrenal gland has also been implicated in the acute photic modulation of GC release [15]; however, the relative importance of this response in the circadian modulation of GC release is unknown.

**Figure 1:**
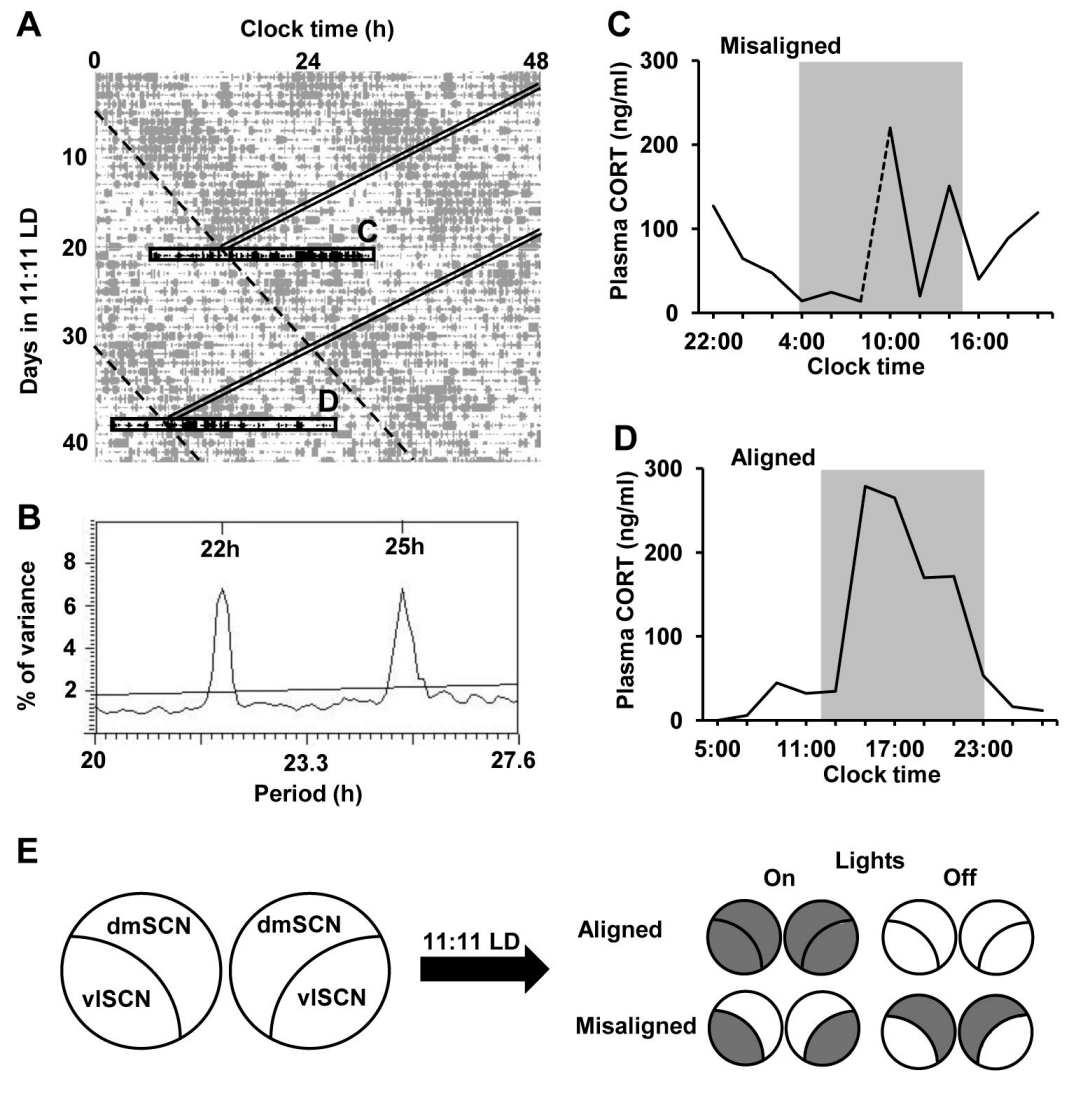
Forced desynchrony disrupts the plasma corticosterone rhythm. A) Double-plotted locomotor activity of a representative forced desynchronized rat. Grey bars represent locomotor activity; double diagonal bands indicate the time of lights-on (*for box “C”*) or time of lights-off (*for box “D”*); dashed diagonal lines indicate the onset of dmSCN-associated locomotor activity bout with the angle set to match the LD-dissociated period indicated in the periodogram (B); black horizontal boxes indicate the 24-h periods during which blood samples were taken every two hours during misaligned (*top, “C”*) or aligned (*bottom, “D”*) phases. B) The X^2^ periodogram analysis of locomotor activity revealing two statistically significant rhythmic components (22 h and 25 h) in the animal shown in (A). C and D) The plasma corticosterone profiles (raw data) determined for the one animal shown in (A) during the days of misalignment and alignment, respectively. The dotted line in (C) represents predicted values between initial (t = 10: 00) and final (t = 8: 00) sampling times. Grey shading indicates time of lights off. E) Schematic diagram of predicted *Per1* expression patterns in the subregions of the SCN at 5.5 h after lights-on or lights-off on days of alignment and misalignment. Predicted *Per1* expression patterns (grey and white for high and low expression, respectively) are based on previous studies [20,22,28].

The SCN is a heterogeneous nucleus; neurons within the ventrolateral (vl) and dorsomedial (dm) SCN express different neuropeptides and show different patterns of efferent and afferent projections [16]. Until now the role of the SCN in the regulation of the HPA axis has been addressed predominantly by lesion studies [1,5,17–19]; however, the role of each subregion cannot be assessed by neuroanatomical lesions mainly because vlSCN efferent fibers course through the dmSCN. Furthermore, knockouts that target gene expression in specific SCN subregions are not available. We have developed a forced desynchrony model in the rat that induces the desynchronization of vlSCN neurons from dmSCN neurons in genetically and neurologically intact rats [20]. When rats are exposed to an 11:11 light-dark (LD) cycle (LD22), they develop two locomotor activity rhythms with differing periods: one rhythm is entrained to the 22 h LD cycle and the other rhythm is dissociated from it and shows a period of ~25 h. We have previously shown that this ~25 h rhythm is not truly free-running, but still weakly coupled to the vlSCN [21]; therefore, we refer to it as the LD-dissociated rhythm. As the two rhythms in the LD22 desynchronized rat come in and out of phase with each other, the animals experience days of alignment, where both activity phases coincide, and days of misalignment, where the activity phase of one rhythm ends as the activity phase of the other begins. These rhythmic outputs parallel ‘clock gene’ expression rhythms within the vl- and dmSCN (Figure 1E). Using this animal model we have shown that the two SCN subregions have different roles in circadian output regulation [20–24]. Here, we use this model to reveal the independent contributions of these two subpopulations of SCN neuronal oscillators to the GC daily rhythm. Furthermore, we test the hypothesis that outputs from these subpopulations modulate adrenal responsiveness to ACTH via a multi-synaptic neural pathway through the splanchnic nerve.

## Materials and Methods

### Ethics Statement

Adult male Wistar rats, purchased from Charles River, were used for all the experiments. All experiments were performed according to the National Institutes of Health Guide for Care and Use of Laboratory Animals and were approved by the Institutional Animal Care and Use Committees at the University of Washington (for experiment 1; IACUC # 4045-01) and the Pacific Northwest Diabetes Research Institute (for experiment 2; IACUC # 106-08).

### Activity Cycles and Internal Desynchronization

Animal activity monitoring and internal desynchronization was carried out as reported previously [20]. Briefly, animals were singly housed either under a 12:12 light-dark cycle (LD24 control animals) or an 11:11 LD cycle (LD22 animals) with 50-150 lux light during the light phase and red light not brighter than 1 lux during the dark phase. LD22 animals were housed under 11:11 for 1-2 months. Locomotor activity was recorded using 2 perpendicular infrared beams crossed through the center of the cage, ~2 cm above the bedding, with beam breaks recorded digitally with ClockLab (Actimetrics, Wilmette, IL). Analysis of activity was carried out using the software “El Temps” (Dr. Antoni Díez-Noguera, University of Barcelona) and the graphs imported into Adobe Photoshop to prepare final figures. Only LD22 animals showing two statistically significant rhythms using X^2^ periodogram analysis of locomotor activity (El Temps) were included in each experiment. Assessment of alignment and misalignment during the forced desynchrony protocol was done by eye-fitting the onset-timing line of the ~25 h rhythm, by at least two independent researchers, and by highlighting the dark phase of the LD cycle, as described previously [20]. Significantly desynchronized animals were grouped into either “aligned” or “misaligned” groups for blood and tissue collection; this did not change their housing or handling, only the time of collection relative to their activity cycles.

### Experimental Protocols

#### Experiment 1: Effect of desynchronization on HPA rhythmic activity

Once animals became desynchronized, they were implanted with silastic catheters. The jugular vein was exposed and raised from the tissue, nicked but not severed, and silastic tubing (I.D. 0.51 mm, O.D. 0.94 mm) was inserted 4.3 cm, with its tip in the right atrium. The catheter was anchored by suturing, threaded under the skin, extruded through the back of the neck, filled with 20µl heparin and 120µg gentamicin/ml saline and plugged until bleeding.

Animals were allowed to recover for at least 6 days, then blood samples (250 µl) were collected through the implanted catheters into 1 ml syringe pre-filled with 7 µl EDTA (60 mg/ml) every 2 h for 24 h. Blood volume was replaced with 35^°^C saline, and the catheter refilled with heparinized saline. Bleeding and volume replacement took less than 3 min per animal, and was done without any type of restrain other than gently holding the animals, which were accustomed to this manipulation from prior handling. Samples were then centrifuged at 4^°^C, 1500 rpm, for 15 min. Plasma was separated and stored at -80^°^C for subsequent processing of corticosterone and ACTH by radioimmunoassay (RIA). After 24 hr collections were complete, animals were allowed to recover for approximately one to two weeks, at which time a second series of sampling was performed. The second round of sampling began when the rats were in the opposite phase (aligned or misaligned) from what they were during the first round of sampling. After the final serial blood collection, animals were sacrificed by decapitation. Brains were dissected immediately and snap-frozen in -30^°^C methyl butane; trunk blood was collected into tubes with 120 µl EDTA (60µg/ml) and centrifuged as above. Plasma and brains were then stored at -80^°^C until processing for an independent study.

#### Experiment 2: Effect of splanchnicectomy on desynchronized HPA rhythm

Under isoflurane anesthesia, all rats underwent unilateral right adrenalectomy; the right adrenal gland was exposed and removed via a dorsolateral incision and right subcostal muscle penetration. Unilateral adrenalectomy was performed to minimize the loss of splanchnic innervation to extra-adrenal tissues that could be affected by splanchnicectomy on the contralateral side. The thoracic splanchnic nerve, proximal to the left adrenal, was then either cut (SPLNX) to sever preganglionic sympathetic and primary afferent fibers, or visualized (Sham), as described in [13]. Briefly, an approximately 2 inch dorsoventral incision was made on the left side of the body just below the rib cage. The adrenal nerve was followed toward the spinal cord to locate the thoracic splanchnic nerve as it exits from the sympathetic chain. The splanchnic nerve was ligated and cut close to the sympathetic chain and proximal to the suprarenal ganglion (SPLNX). In Sham animals, the nerve was visualized, but not manipulated. Animals were allowed to recover from surgery for at least 7 days before being placed on the desynchronization protocol and then catheterized. After another 6-7 days of recovery, animals were then sampled over a 24 h period as described in experiment 1. After the final serial blood collection, animals were sacrificed by decapitation, and brains and trunk blood were collected and stored as described above. Left adrenal glands were removed, cleaned and weighed, and the absence of the right adrenal was verified [13]. The left adrenals were then placed in Zamboni’s fixative (0.2% picric acid and 2% paraformaldehyde in 0.16 M phosphate buffer, 4^°^C) for at least 24 h, with subsequent placement into sucrose cryoprotectant (20% sucrose in 0.05 M phosphate-buffered saline (PBS), 4^°^C) for at least 24 h, after which time they were frozen at -80^°^C until subsequent immunohistochemical detection of nerve fibers. Plasma samples were subsequently measured for ACTH and corticosterone by RIA.

### Determination of Plasma Hormones

Plasma ACTH was determined by RIA with ^125^I labeled ACTH (DiaSorin, Stillwater, MN) as described previously [25]. The intra-assay and inter-assay coefficients of variation (CVs) for ACTH were 5.5% and 4.6%, respectively. Plasma corticosterone was determined by an RIA kit (ICN Biochemical, Costa Mesa, CA). The intra-assay and inter-assay CVs for corticosterone were 7.6% and 13.3%, respectively.

### Immunolabeling of Nerve Fibers

Denervation of the adrenal was verified by labeling for calcitonin gene-related peptide (CGRP), found in primary afferent fibers in the adrenal cortex, and the vesicular acetylcholine transporter (VAChT), found in pre-ganglionic nerve terminals innervating the adrenal medulla [13]. Both of these fiber types are carried primarily through the thoracic splanchnic nerve, therefore, absence of their labeling suggests successful nerve transection and reduced innervation of the adrenal.

Post-fixed adrenals were cyrosectioned (20µm), washed free-floating 3 X 10 min, in PBS (0.01M) and then incubated for 30 min with 0.3% H_2_O_2_ in PBS to block endogenous peroxidase activity. After rinsing again for 3 X 10 min in PBS, sections were incubated for 1 h in PBS with 5% normal donkey serum and 0.2% Triton X-100. Alternating sections were then incubated overnight at 4^°^C with either rabbit anti-CGRP primary antibody (1:20,000; Immunostar, Hudson, WI; cat # 24112) or goat anti-VAChT primary antibody (1:40,000; Immunostar; cat # 24286); sections used for VAChT labeling underwent citrate (5mM) pre-treatment for 30 min at 100^°^C prior to incubation with primary antibody to enhance antigen detection. The next day, CGRP- and VAChT-immunoreacted sections were incubated for 1 h at room temp in either donkey anti-rabbit biotinylated secondary (1:250; Jackson ImmunoResearch Laboratories, West Grove, PA; cat 711-065-152) or donkey anti-goat biotinylated secondary antibody (1:250; Life Technologies, Grand Island, NY; cat # D20698), respectively. Labeling was visualized using a Vectastain ABC kit (Vector Labs, Burlingame, CA) followed by incubation in diaminobenzidine with nickel (Vector Labs). Sections were washed, dehydrated and then cover-slipped with permount (Sigma Chemical Co., St. Louis, MO). All slides were examined for labeling using bright field microscopy by two researchers blinded to the splanchnic nerve treatment.

### Statistics

To analyze plasma hormone profiles, data from individual animals were first smoothed using a 3-point running average, and then plotted as means ± SEM. We then used a sine wave analysis to determine the effect of group (aligned vs. misaligned and SPLNX vs. Sham) on the peak of the plasma hormone profiles. We fit the plasma hormone profile with a sinusoidal function having a constant bias: *h* (t) = β_0_ sin (β_1_ *t*+β_2_) + β_3_ by minimizing the mean square error. The phase β_2_ determines the time of the peak of the plasma hormone profile. The time of the peak is found using the formula t peak = (5π/2 -β_2_)/β_1_. For each condition, 2000 bootstrap samples of the residuals in the regression were used to estimate distributions and standard errors for the model coefficients [26]. A two-sample Kolmogorov-Smirnov (KS) test was then used to determine differences between the phases and peak amplitudes of the sine functions fitting the plasma hormone profile under different conditions.

## Results

### Misalignment of the dmSCN and vlSCN locomotor activity rhythms disrupts the circadian rhythm of ACTH and corticosterone secretion

In rats on a 12:12 LD cycle, the circadian peak in ACTH and corticosterone secretion occurs just before the onset of locomotor activity, which is around, or just prior to the onset of lights-off [27]. We have previously shown, that rats under LD22 desynchrony exhibit both 22 h and ~25 h locomotor activity rhythms [20,23,28]. The goal of this experiment was to use the LD22 forced desynchrony model to determine whether the rhythmic activity of the HPA axis is associated with the 22 h rhythm, the ~25 h rhythm or both, and therefore how oscillators in the vl- and dmSCN contribute to the plasma ACTH and corticosterone daily rhythm.

**Figure 2:**
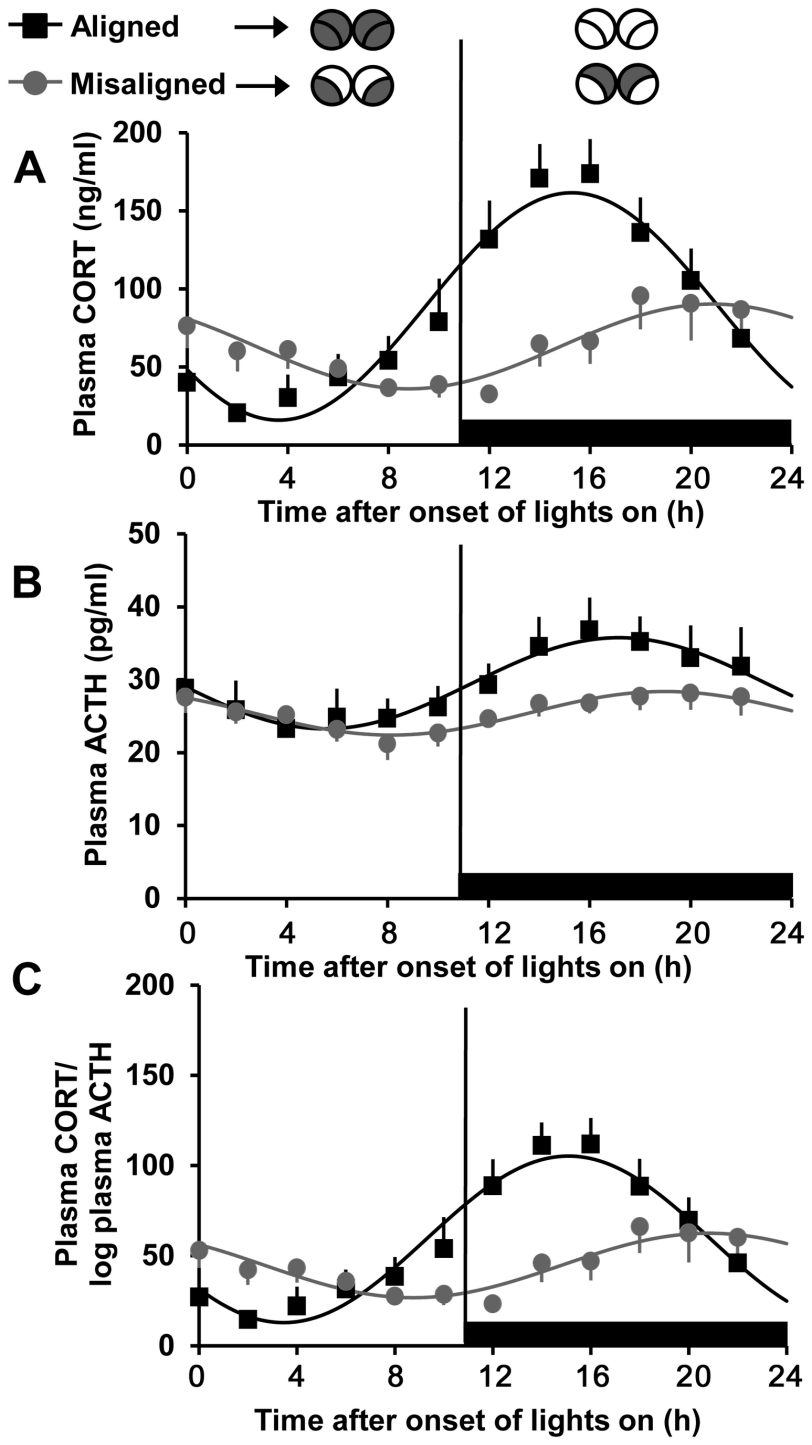
The plasma corticosterone rhythm is disrupted on days of misalignment. Plasma corticosterone (A), ACTH (B) and corticosterone/log ACTH (C) on aligned (*black*) and misaligned (*grey*) days. Black horizontal bars denote times when lights are off. Illustrations (*top*), as in Figure 1E, show predicted *Per1* expression patterns in the subregions of the SCN at the midpoint of the lights-on and lights-off on days of alignment and misalignment. Data are means ± SEM and fitted with a sine wave function (see materials and methods). n = 5; the same animals make up both aligned and misaligned groups.

In experiment 1, LD22 resulted in desynchronization of locomotor activity rhythms with two statistically significant rhythmic components (22 h and ~25 h) within each individual (Figure 1A and B). Desynchronized rats exhibited significant plasma corticosterone rhythms on both aligned [sine parameters: 72.86, 0.27, 3.73, 88.79 (β_0_, β_1_, β_2_, β_3_), P < 0.0001 for each, R^2^ = 0.958, n = 5; Figures 1D and 2A] and misaligned (sine parameters: 27.16, 0.26, 2.43, 63.17, P < 0.0001 for each, R^2^ = 0.887, n = 5; Figures 1C and 2A) days; however, the peak of the corticosterone rhythm was reduced on misaligned days compared to aligned days (two-sample KS test: p < 0.0001). The phase of the corticosterone peak was also significantly delayed on days of misalignment compared to days of alignment (20.9 h vs. 15.3 h, two-sample KS test: p < 0.0001). Desynchronized rats also exhibited significant plasma ACTH rhythms on both aligned (sine parameters: 6.25, 0.27, 3.22, 29, 52, P < 0.0001 for each, R^2^ = 0.96, n = 5) and misaligned (sine parameters: 2.99, 0.29, 2.35, 25.40, P < 0.0001 for each, R^2^ = 0.922, n = 5) days (Figure 2B). Like plasma corticosterone, the peak of the ACTH rhythm was reduced and the phase was delayed (19.0 h vs. 17.2 h) on misaligned days compared to aligned days (two-sample KS test: p < 0.0001 for both parameters). A comparison of the phase relationship between the corticosterone and ACTH rhythms showed significant differences on both aligned and misaligned days as well (two-sample KS test: p < 0.0001); on days of alignment the peak in ACTH occurred after the peak in corticosterone, whereas on days of misalignment the peak in ACTH preceded the peak in corticosterone.

The finding that the phases of the plasma corticosterone and ACTH rhythms differed both on days of alignment and misalignment, suggests that changes in adrenal sensitivity may influence the corticosterone rhythm more than absolute plasma concentrations of ACTH. To better visualize this, plasma corticosterone divided by the log of plasma ACTH, and index of adrenal responsiveness to ACTH [13,29,30], is shown in Figure 2C. The ratio of plasma corticosterone to the log of plasma ACTH varied over time similarly to that observed for plasma corticosterone, on both aligned (sine parameters: 46.17, 0.27, 3.78, 59.14, P < 0.0001 for each, R^2^ = 0.96) and misaligned days (sine parameters: 17.87, 0.26, 2.44, 44.65; P < 0.0001 for each, R^2^ = 0.873), suggesting a robust change in sensitivity. There was also a significant difference in phase (two-sample KS test: p < 0.0001) and peak amplitude (two-sample KS test: p < 0.0001) between days of alignment and days of misalignment.

### Splanchnicectomy reduces the peak and advances the phase of the HPA rhythm during forced desynchrony

Changes in the plasma corticosterone rhythm were dissociated from the changes in plasma ACTH in desynchronized rats; therefore, we speculated that different profiles of corticosterone might emerge from changes in adrenal sensitivity to ACTH. Because the splanchnic nerve has been shown to regulate the sensitivity of the adrenal gland to ACTH [11–13,31], splanchnicectomy was performed on forced desynchronized animals to determine whether the vlSCN and/or dmSCN contribute to the regulation of plasma corticosterone via adrenal innervation.

**Figure 3:**
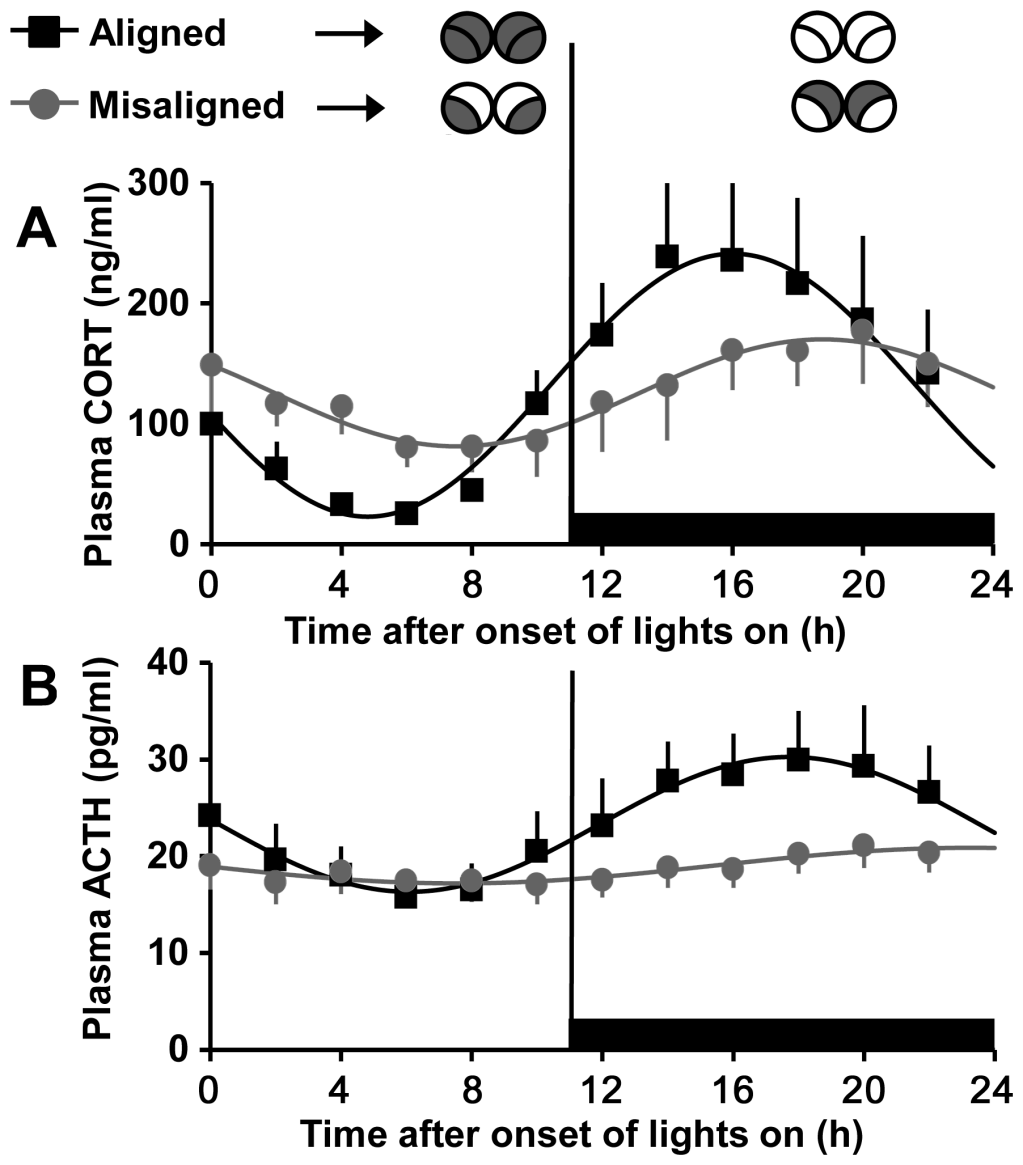
Plasma corticosterone and ACTH rhythms are disrupted in Sham-operated forced desynchronized rats. Plasma corticosterone (A) and ACTH (B) in Sham treated rats on aligned (*black*) and misaligned (*grey*) days. Black horizontal bars denote times when lights are off. Illustrations (*top*) show predicted *Per1* expression patterns, as in Figure 2. Data are means ± SEM and fitted with a sine wave function (see materials and methods). n= 6 rats/group.

In Sham-treated rats, plasma corticosterone (Figure 3A) and ACTH (Figure 3B) profiles were similar to that observed in experiment 1; sine wave analysis revealed significant rhythms on days of alignment and misalignment for both plasma corticosterone (sine parameters for days of alignment: 109.16, 0.28, 3.37, 132.19, P < 0.0001 for each, R^2^ = 0.985; days of misalignment: 44.42, 0.28, 2.60, 125.83, P < 0.0001 for each, R^2^ = 0.956; n = 6) and ACTH (sine parameters for days of alignment: 6.97, 0.27, 3.07, 23.30, P < 0.0001 for each, R^2^ = 0.986 ; days of misalignment: 1.84, 0.20, 3.20, 19.04, P < 0.0025 for each, R^2^ = 0.853; n = 6). As in experiment 1, both the corticosterone and ACTH peaks were reduced and phase delayed on misaligned days compare to aligned days (two-sample KS test: p < 0.0001 for all comparisons). The plasma corticosterone peaks were elevated on both days of alignment and misalignment in Sham-treated rats compared to rats that did not undergo laparotomy from experiment 1 (two-sample KS test: p < 0.0001 for both comparisons); however, plasma ACTH peaks were reduced (two-sample KS test: p < 0.0001 for both comparisons).

**Figure 4:**
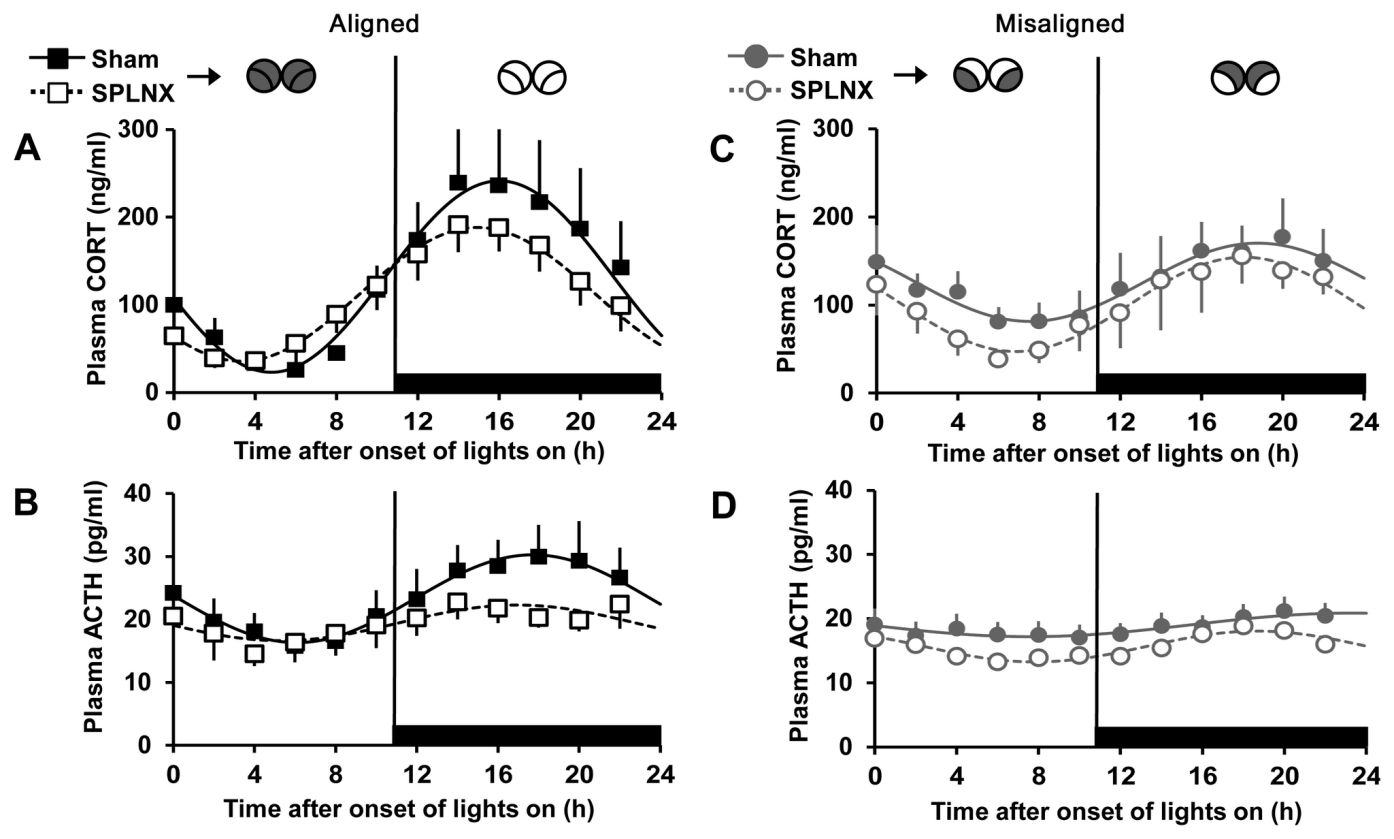
Splanchnicectomy reduces and phase advances the peak of the plasma corticosterone and ACTH rhythms. Plasma corticosterone (A and C) and ACTH (B and D) in Sham (*solid lines*) and SPLNX (*dotted line*) rats on aligned (*black*) and misaligned (*grey*) days. Black horizontal bars denote times when lights are off. Illustrations (*top*) show predicted *Per1* expression patterns, as in Figure 2. Data from Sham animals are the same as those presented in Figure 3. Data are means ± SEM and fitted with a sine wave function (see materials and methods). n= 6-7 rats/group.

In SPLNX-treated rats, sine-wave analysis revealed that plasma corticosterone and ACTH exhibited a significant rhythm on both days of alignment (sine parameters for corticosterone: 76.25, 0.27, 3.82, 111.96, P < 0.0001 for each, R^2^ = 0.995; sine parameters for ACTH: 2.83, 0.27, 3.28, 19.46, P < 0.004 for each, R^2^ = 0.684; n = 8; Figure 4A and B) and misalignment (sine parameters for corticosterone: 53.53, 0.28, 2.79, 100.74, P < 0.0001 for each, R^2^ = 0.973; sine parameters for ACTH: 2.4, 0.29, 2.44, 15.67, P < 0.0001 for each, R^2^ = 0.92; n = 7; Figure 4C and D). Comparisons of Sham vs. SPLNX rats showed that transection of the thoracic splanchnic nerve reduced the peak and advanced the phase of the plasma corticosterone rhythm on both days of alignment and misalignment (two-sample KS test: p < 0.0001 for all comparisons). Analysis of plasma ACTH from Sham vs. SPLNX rats revealed that transection of the thoracic splanchnic nerve also reduced the peak and advanced the phase of the plasma ACTH rhythm on both days of alignment and misalignment (two-sample KS test: p < 0.0001 for all comparisons).

**Figure 5:**
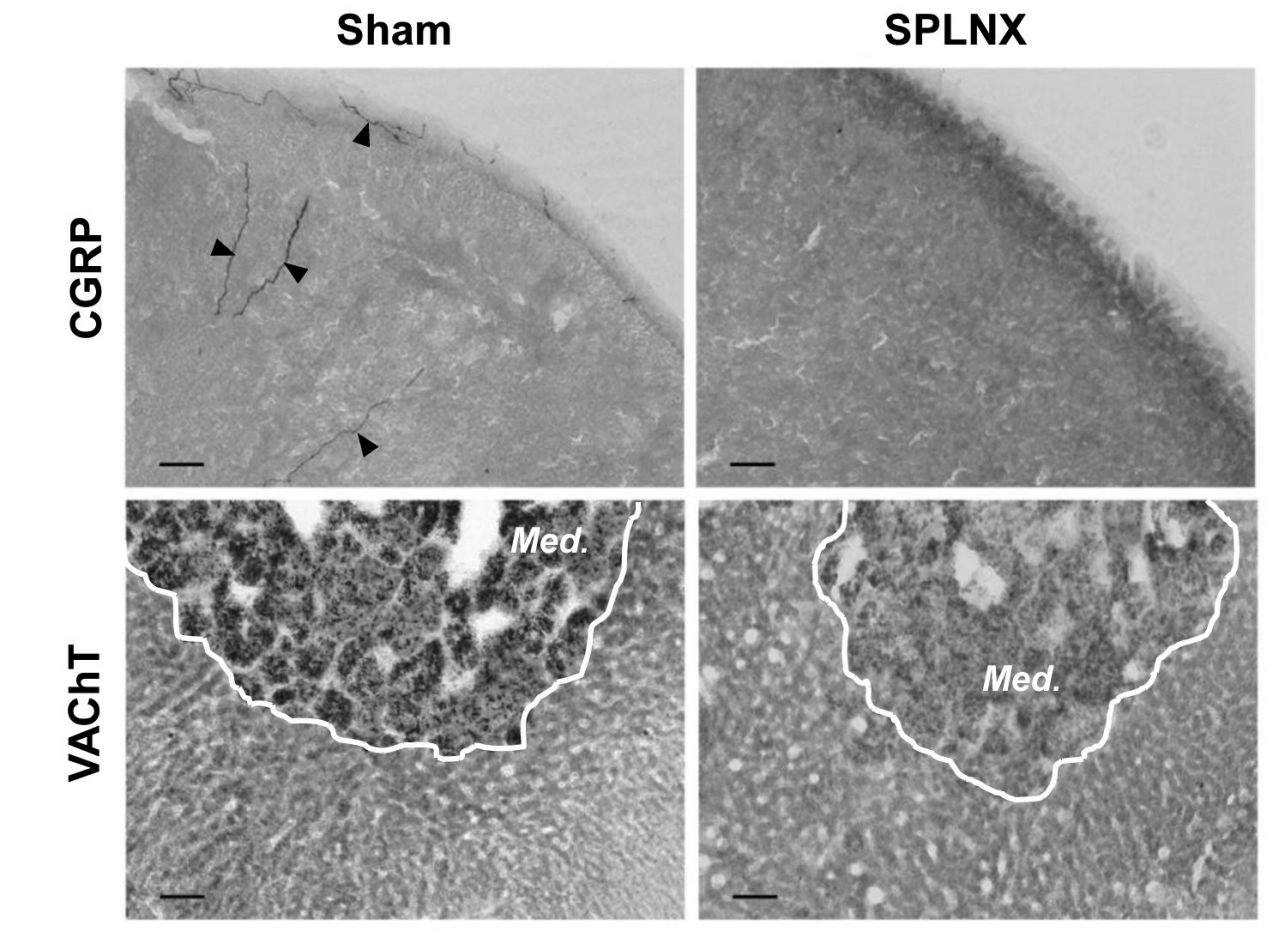
Splanchnicectomy effectively removed primary afferent and pre-ganglionic sympathetic innervation of the adrenal. Immunohistochemical labeling for calcitonin gene-related peptide (CGRP; *top*) and the vesicular acetylcholine transporter (VAChT; *bottom*) in representative adrenal sections from Sham (*left*) and SPLNX (*right*) treated rats. CGRP-positive fibers (arrows) are found in capsule and cortex of adrenals from Sham rats, whereas VAChT-positive fibers are found in the medulla (*outlined by white line; Med*). Note labeling for each marker is absent in adrenals from SPLNX rats, confirming the removal of splanchnic innervation. Bars = 100 µm.

Adrenals from Sham nerve-transected animals exhibited CGRP-positive fibers in the capsule, cortex and medulla and VAChT-positive fibers in the medulla (Figure 5); these fiber types were largely absent in adrenals from animals that underwent nerve-transection.

## Discussion

The role the SCN plays in the daily release of GC has been well documented [for review, see 2]; however, the independent contributions of SCN subregions to the GC rhythm remain unclear. Classic neuroanatomical lesion studies to address this question are challenging because of the difficulty to individually target the subregions and because vlSCN efferent fibers course through the dmSCN [16]. The forced desynchronized rat represents a unique model in which the output of the vl- and dmSCN subregions to the HPA axis can be assessed in a genetically, neurologically and pharmacologically intact animal.

Here we have shown that on days of alignment, rats placed on an LD22 desynchrony protocol exhibit a plasma corticosterone rhythm with a peak that occurs approximately 4 h after the onset of lights-off. Although the amplitude of this rhythm is similar, its phase is delayed compared to what has been reported in rats on a 12:12 LD cycle [2]. This could in part be due to the fact that even when the locomotor activity bouts are in phase with each other during aligned days, the relative phase of endocrine rhythms to locomotor activity may not ever reach a normal phase during the desynchrony protocol. On days of misalignment, when the 22 h (light entrained) and ~25 h (LD-dissociated) components of the locomotor rhythm are maximally out of phase (~180^°^) with each other, plasma corticosterone shows a lower amplitude rhythm with a peak onset that occurs approximately 9 h after the onset of lights-off and is not phase-locked to the onset of either locomotor activity rhythmic component. Because the 22 h and ~25 h locomotor activity rhythms are associated with rhythmic clock gene expression within the vl- and dmSCN, respectively [20,22,28], our data suggest that the characteristic plasma corticosterone rhythm that occurs in rats on a 12:12 LD cycle is not the result of signaling from oscillators within either the vl- or dmSCN alone, but instead of the combined output from both subregions.

We have previously shown that on days of alignment, both subregions of the SCN express high levels of the clock gene *Per1* during the light phase and low levels during the dark phase, and that these expression patterns correlate with resting and active locomotor states, respectively [20,22,28]. However, on misaligned days, the pattern of expression is 180^°^ out of phase between the two subregions; the dmSCN shows elevated *Per1* expression during the coinciding dark phase and ~25 h subjective day, whereas the vlSCN shows elevated *Per1* expression during the coinciding light phase and ~25 h subjective night. Because *Per1* expression has been linked to neuronal activity and synaptic transmission in the SCN (for review, see 32, measuring plasma corticosterone on days of misalignment can provide insight into how the activity of vl- and dmSCN neuronal oscillators presumably sustain the corticosterone rhythm. Clearly, high *Per1* expression in both subdivisions of the SCN, as occurs during the lights-on/subjective day period on aligned days, correlates with the nadir of the plasma corticosterone rhythm. These data, along with those from SCN lesion studies [5] suggest that daytime activity in both subregions leads to inhibitory output to the HPA axis. In contrast, activity of either one of the SCN subdivisions alone, as occurs during days of misalignment, correlates with plasma corticosterone levels that are above the nadir levels but below peak levels of aligned days, suggesting that output from either subdivision has a partial inhibitory effect. Furthermore, corticosterone levels decrease over time during the lights-on/subjective night phase and increase over time during the lights-off/subjective day phase on days of misalignment. These seemingly opposing responses are in agreement with the proposed inhibitory and stimulatory signals from the SCN that overlap over the course of the light period in rats on a 12:12 LD cycle [2,6], conditions in which the vlSCN *Per1* expression is high during the early light phase and decreases throughout the light phase, probably reducing inhibitory output to the HPA axis [33]. In a series of elegant studies, it was shown that inhibition, mediated by vasopressin release from the SCN—predominantly from dmSCN neurons—in the first half of the light period, is likely responsible for the nadir of the corticosterone daily rhythm, and that release from this inhibition during the second half of the light period, along with increasing stimulatory output, is responsible for the circadian rise at the beginning of the dark period [6,27,34]. Together, our data support this contribution of the dmSCN to the corticosterone rhythm, and also suggest that signals from the vlSCN are important for the daytime inhibition of the HPA axis. Although vasoactive intestinal peptide (VIP), which is predominantly found in the vlSCN, has been shown to stimulate HPA activity [35,36], our findings would not support the role of the vlSCN as a stimulatory influence on circadian corticosterone secretion.

The vlSCN receives direct neural input from the retina and is believed to convey information about the external environment, such as light cues, in part through efferent neural pathways to the dmSCN. Accordingly, neural activity and clock gene expression is highest in both SCN subregions during the light phase in rats under LD24 [33]. The role that photic information plays in controlling GC rhythms remains unclear. In two independent studies, photic stimulation has been shown to change GC levels in an SCN-dependent but ACTH-independent manner. In these studies, light pulses were given at either the beginning of the dark phase to rats [14] or the beginning of subjective night to mice [15]. In the first study, light exposure resulted in a rapid (within 5 min) decrease in plasma corticosterone [14] whereas, in the second study, light resulted in a longer-term (60 min after the light pulse) increase in plasma corticosterone [15]. Our data are consistent with the findings that photic information, conveyed through the vlSCN, results in an inhibition in corticosterone secretion, although this response could depend on circadian phase and be different between species.

Interestingly, in both of the studies mentioned above light induced changes in corticosterone did not correlate with changes in plasma ACTH. Because the inhibitory response to light was rapid, and the delayed stimulatory response was abolished by denervation of the adrenal, it has been hypothesized that a multisynaptic, sympathetic neural pathway from the SCN to the adrenal mediates these responses. Tract-tracing studies have defined a pathway through which vasopressinergic and VIPergic SCN neurons project to pre-autonomic neurons in the dorsal region of the PVN, which in turn innervate neurons in the intermediolateral nucleus of the spinal cord that project to the adrenal cortex [14]. Such a neural pathway has not only been implicated in driving the adrenal responses to light, but also in mediating changes in sensitivity to ACTH that occur during the rising phase of the GC daily rhythm [11,12]. Although the GC daily rhythm is robust, ACTH rhythms have been shown to be low in amplitude, or non-existent [6–10], suggesting that the circadian release of GC could be independent of circadian ACTH release. Accordingly, we have recently confirmed both a low amplitude ACTH rhythm and a high amplitude cortisol rhythm in hamsters exhibiting normal circadian activity [37]. In contrast, split hamsters, which show a circa-12 h rhythm of locomotor activity, exhibit two peaks of cortisol in the absence of concomitant ACTH release, clearly showing that circadian release of GCs can occur in the absence of circadian ACTH release. In the present study, we also observed changes in plasma corticosterone without concomitant changes in ACTH. The ratio of plasma corticosterone to the log of plasma ACTH, and index of adrenal responsiveness to ACTH, varied over time on days of alignment or misalignment, suggesting that either changes in adrenal sensitivity to ACTH throughout the day, or ACTH-independent factors contribute to the plasma corticosterone rhythm. Moreover, the peak ratio of the plasma corticosterone to the log of plasma ACTH was reduced and phase delayed on days of misalignment compared to days of alignment. These data suggest that the difference in peak plasma corticosterone values between aligned and misaligned animals, and the timing of the peaks, differ because of the independent effects of the vl- and dmSCN on changes in adrenal sensitivity to ACTH. The importance of adrenal cortical innervation in enhancing the evening rise in corticosterone by increasing the response to low plasma ACTH has been demonstrated by cutting the thoracic splanchnic nerve, which supplies both pre- and post-ganglionic sympathetic, as well as primary afferent fibers to the gland [11]. We utilized the same approach here to determine if the disparities we observed between plasma corticosterone and ACTH could be explained by contributions of the splanchnic nerve.

Based on previous studies, we anticipated that on aligned days, splanchnicectomy would result in a decrease in plasma corticosterone during the lights-off/active phase of the rhythm [11,31,38], and possibly an increase in plasma corticosterone during the lights-on/rest phase of the rhythm [39]. Our data are consistent with this prediction, as transecting the splanchnic nerve reduced the amplitude and phase advanced the plasma corticosterone rhythm on days of alignment. We saw the same effect of splanchnicectomy on days of misalignment. Interestingly, the greatest effect of cutting the splanchnic nerve appeared to occur on days of alignment when the vl- and dmSCN are inactive (lights-off/active phase) and on days of misalignment when only the vlSCN is active (lights on/active phase), suggesting the nerve has a stimulatory output to the adrenal when the dmSCN is inactive, which is inline with the literature. Interestingly, these effects of splanchnicectomy were also observed on the plasma ACTH rhythm, suggesting that cutting the splanchnic nerve had an effect on the anterior pituitary. This result may reflect an interaction between desynchrony and splanchnicectomy that results in feedback on the anterior pituitary. This feedback could either be hormonal or neural, as the thoracic splanchnic nerve carries afferent, as well as efferent fibers. Finally, peak corticosterone levels were higher, whereas, peak ACTH levels were lower, in experiment 2 compared to experiment 1. These data suggest an increase in adrenal sensitivity or ACTH-independent factors contributing to elevated plasma corticosterone in experiment 2. It is possible that the two extensive surgical procedures performed on rats in experiment 2 resulted in higher baseline corticosterone due to stress, and that this response was mediated by the splanchnic nerve. A similar response to surgical stress has been previously described [39]; however, this effect was observed at one and two days after surgery, but resolved by five days after surgery. Blood samples in the current study were collected no sooner than three weeks after the splanchnicectomy surgery and seven days after the catheterization surgery; therefore, it is unlikely our animals were experiencing the effects of surgical stress at the time of collection. The second experiment was done with a different cohort of animals and in a different animal facility, which could account for the differences in overall levels of stress hormones; nevertheless, the effects of circadian desynchrony on the HPA axis were the same in experiments 1 and 2.

**Figure 6:**
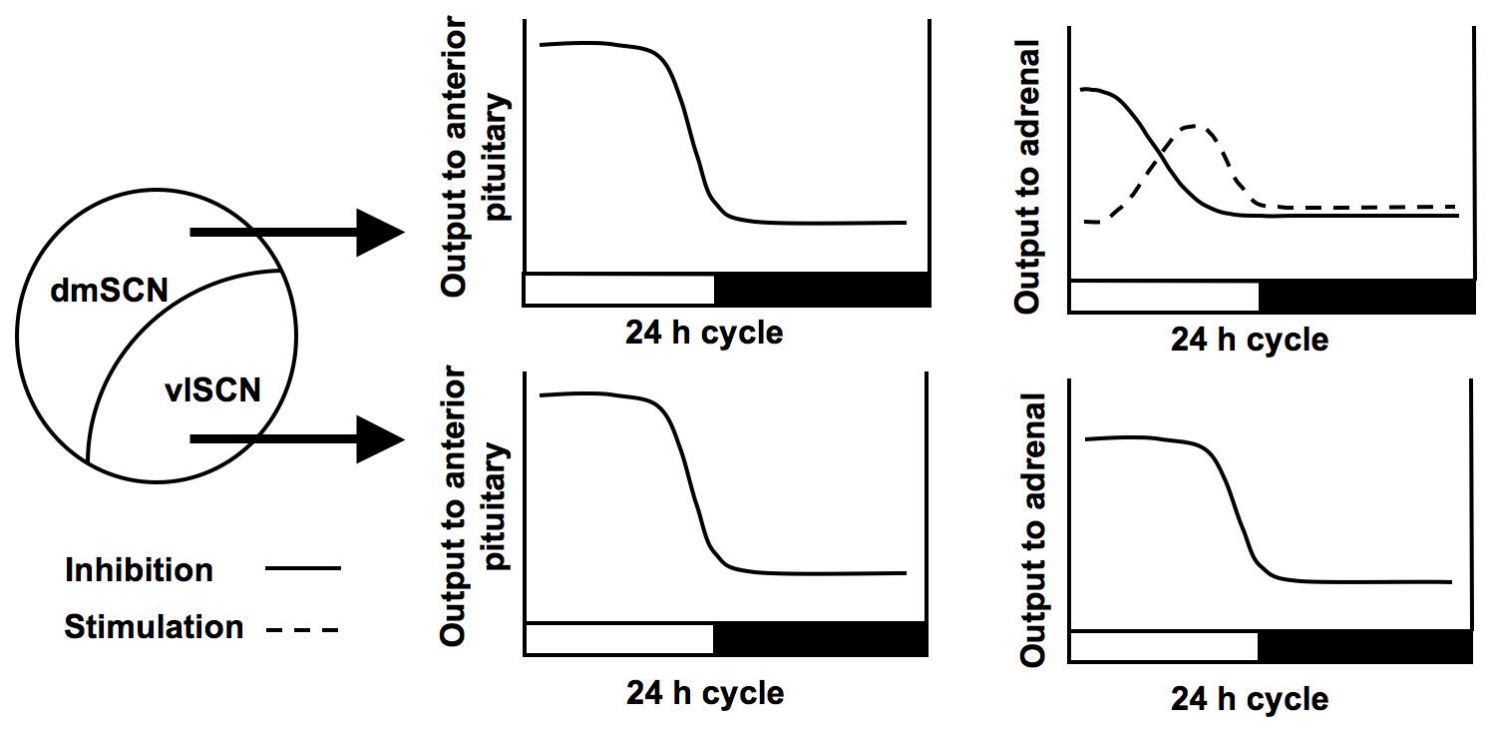
Model of output from SCN subregions to the anterior pituitary and adrenal glands. During the light phase, the dmSCN and vlSCN inhibit ACTH release from the pituitary, while the dmSCN slowly increases plasma corticosterone through either decreasing inhibitory, or increasing stimulatory, output to the adrenal. At the same time, the vlSCN supplies inhibitory output to the adrenals over the light period, keeping plasma corticosterone low until the onset of the dark phase, at which time the pituitary is released from inhibition, resulting in a heightened plasma corticosterone response to ACTH.

Based on the findings presented here, we present a model in which the daily rhythm of plasma corticosterone is determined by the combined input of neural oscillators in both the vl- and dmSCN (Figure 6). During the light (rest) phase of a 12:12 LD cycle, the dmSCN and vlSCN together inhibit ACTH release, whereas the dmSCN slowly enhances the release of plasma corticosterone through a mechanism — independent of an ACTH increase—that may be mediated by the turning-on, and subsequent turning-off, of vasopressinergic efferent pathways. At the same time, the vlSCN suppresses the effects of the dmSCN until the onset of the dark phase, at which time the pituitary and adrenals are released from inhibition, resulting in a heightened plasma corticosterone response to ACTH. Whether or not mediated through the splanchnic nerve, it seems reasonable to suggest that the dmSCN increases plasma corticosterone towards the end of the light phase by either 1) enhancing adrenal responsiveness to ACTH, or 2) through an ACTH-independent mechanism. Future studies testing adrenal sensitivity to exogenous ACTH during aligned vs. misaligned days of desynchrony could address the first hypothesis. Although the second hypothesis is more challenging to elucidate, investigating the effects of forced desynchrony on adrenal clock gene rhythms may shed light on how the SCN may drive corticosterone secretion through ACTH-independent mechanisms [40,41]. It is also possible that changes in corticosterone clearance out of the plasma could account for the differences in plasma corticosterone independent of concomitant changes in ACTH we that observed. Finally, we should also consider the potential for non-SCN mediated mechanisms contributing to the plasma corticosterone rhythms we observe during desynchrony. One such factor could be changes in food intake, as feeding has been shown to entrain plasma corticosterone rhythms independently of the SCN [42]. Although we have not measured food intake in our desynchronized rats, it is possible that, like locomotor activity, bouts of feeding are more evenly distributed on days of misalignment, leading to the dampened corticosterone rhythm we observe.

The forced desynchronized rat is the only model of circadian internal desynchronization for which the neural bases of the misalignment of circadian rhythms is known. In humans, internal desynchronization of circadian rhythms is a typical outcome of exposure to temporal challenges such as jetlag or nocturnal shift-work [43–45]. Interestingly, the GC profiles found during days of alignment and misalignment we report in forced desynchronized rats are remarkably similar to those found in forced desynchronized human subjects [46] and suggest that the misalignment of hormonal profiles under these temporal challenges could also emerge from the desynchronization of subpopulations of neuronal oscillators within the SCN. Circadian misalignment also results in insulin resistance and hyperglycemia in humans [46]; considering the known peripheral effects of GCs, the authors of this study hypothesized that disruption of the GC rhythm by desynchrony could be a contributing factor. The rodent model of desynchrony presented here may represent an ideal system in which to study the effects of HPA dysregulation on the development of metabolic disorders, such as obesity and diabetes, which are prevalent in shift workers [47–49].
